# Cardiometabolic Index (CMI) and Visceral Adiposity Index (VAI) Highlight a Higher Risk of Metabolic Syndrome in Women with Severe Obesity

**DOI:** 10.3390/jcm12093055

**Published:** 2023-04-23

**Authors:** Stefano Lazzer, Mattia D’Alleva, Miriam Isola, Maria De Martino, Diana Caroli, Adele Bondesan, Alice Marra, Alessandro Sartorio

**Affiliations:** 1 Department of Medicine, University of Udine, 33100 Udine, Italy; 2 School of Sport Science, University of Udine, 33100 Udine, Italy; 3 Istituto Auxologico Italiano, IRCCS, Experimental Laboratory for Auxo-Endocrinological Research, 28824 Piancavallo-Verbania, Italy; 4 Istituto Auxologico Italiano, IRCCS, Experimental Laboratory for Auxo-Endocrinological Research, 20145 Milan, Italy

**Keywords:** obesity, metabolic syndrome, anthropometry visceral adiposity index, cardiometabolic index, females

## Abstract

Recent evidence shows that simple and inexpensive anthropometric measurements can be used to identify, at an early stage, women with obesity at increased risk of developing metabolic syndrome (MetS). Thus, the aim of this study was to compare the accuracy of five different indexes of adiposity and/or body composition in identifying MetS in a group of 876 women (mean age ± SD: 52.1 ± 13.8 years; body mass index (BMI): 43.6 ± 6.1 kg m^−2^). The following indexes were determined for each subject: waist-to-hip ratio (WHR), waist-to-height ratio (WtHR), body mass fat index (BMFI), visceral adiposity index (VAI), and cardiometabolic index (CMI). Overall, the presence of MetS was detected in 544 patients (62%). Pearson correlation coefficients were calculated to evaluate the relationships between body composition indexes and metabolic characteristics of the women. Receiver operating characteristic (ROC) analysis was used to determine the best predictor for each adiposity index among metabolic risk factors. The ROC analysis showed VAI (AUC = 0.84) and CMI (AUC = 0.86) showed the best performance in predicting MetS. Differences were found between the ROC area of CMI and VAI with all other indexes (*p* < 0.05). The optimal cutoff point for early diagnosis of MetS was >0.92 for WHR, >0.76 for WtHR, >30.1 kg m^−1^ for BMFI, >1.94 for VAI, and >0.84 for CMI. In addition, VAI and CMI were the most sensitive and specific indexes compared with other indexes. In conclusion, VAI and CMI represent the most useful and reliable indexes to be used for detecting MetS in women suffering from obesity in clinical practice.

## 1. Introduction

In the last decades, the number of obese adults has increased worldwide [1], and the number of people with metabolic syndrome (MetS) is also increasing [2]. There is a direct association between MetS and obesity (i.e., specifically central obesity), which is considered one of the MetS components, according to the International Diabetes Federation (IDF) criteria [3]. Although the diagnosis of MetS is generally more common in male adults than females, this pattern appears to reverse after menopause, when women show a progressive increase in MetS over the years [4]. Indeed, during the menopausal transition, MetS may contribute to doubling the risk of cardiovascular disease while increasing all-cause mortality by 1.5-fold, and it has been associated with cognitive decline and dementia [5]. Thus, to avoid high-cost investigation, recent evidence shows that simple and inexpensive anthropometric measurements can be used to identify, at an early stage, women with obesity at risk of developing a MetS [6].

Body mass index (BMI) is the most used index in clinical practice to predict the risk of developing a MetS [7]. However, BMI does not discriminate between fat-free mass (FFM), fat mass (FM), and its distribution [8]. Since the amount of fat mass and its distribution are closely linked to MetS etiology [9], other indexes based on these parameters have subsequently been developed. These include indexes such as waist-to-hip ratio (WHR) [10], waist-to-height ratio (WtHR) [11], and body mass fat index (BMFI) (i.e., BMI adjusted for body composition and waist circumference) [12]. Previous studies suggested that WtHR is a good predictor of MetS risk factors in children and adolescents with obesity [12] and in a large group of women with obesity [13] compared with BMFI. However, there is no general agreement on which of these indexes has performed best so far.

The diagnosis of MetS integrates anthropometric and biochemical parameters [3]. Therefore, other predictive indexes for MetS have been developed: the visceral adiposity index (VAI) [14], which is based on two anthropometric parameters (waist circumference, WC, and BMI) and two biochemical factors (triglyceride, TG, and high-density lipoprotein, HDL) and the cardiometabolic index (CMI) which integrates marker of adiposity (i.e., WtHR) with the TG/HDL-C ratio [15]. The above two indexes have been shown to predict MetS more accurately in children and adolescents with severe obesity (Radetti et al., 2021), as well as among obese adults [16], than other previously described indexes. However, to our knowledge, no study has compared the reliability of VAI and CMI in detecting MetS in a cohort of women with obesity. Since the metabolic status of women with obesity worsens over the years, early detection of MetS is critical for developing an effective intervention to prevent the adverse health outcomes associated with MetS, such as cardiovascular disease (CVD) and type 2 diabetes (DM).

Therefore, the aim of the present study was to evaluate the capacity of five indexes (WHR, WtHR, BMFI, VAI, and CMI) to identify the presence of MetS in a large group of women with obesity. Since WHR, WtHR, and BMFI consider only anthropometric measures, whereas VAI and CMI integrate anthropometric and biochemical parameters, we hypothesized that VAI and CMI would be the most useful and reliable indexes for detecting MetS in a large group of women with obesity.

## 2. Study Design and Methods

### 2.1. Subjects

A retrospective cohort study based on 876 women (aged 52.1 ± 13.8 (range 18–83) years) with severe obesity (BMI 43.6 ± 6.1 (range 30.7–72.9) kg m^−2^)), consecutively hospitalized for a multidisciplinary 3-week body weight reduction program at the Division of Metabolic Diseases, Istituto Auxologico Italiano, Piancavallo, Verbania, Italy, between April 2017 and April 2019 was performed. All subjects had a full medical history and physical examination, with routine hematology and biochemistry screens and urine analysis. All subjects were Caucasian. All of the subjects suffered from essential obesity, other genetic, organic, endocrine, or iatrogenic forms having been excluded. None of the subjects had taken weight loss drugs during the 12 months before enrollment into the present study. The study was approved by the Ethics Committee of Istituto Auxologico Italiano, IRCCS, (Milan), Italy (ethical committee code of approval: 2022_03_01_06; research project code: 01C215; acronym: VAIOB). The study was performed in accordance with the Declaration of Helsinki and with the 2005 Additional Protocol to the European Convention of Human Rights and Medicine concerning Biomedical Research. At the admission to our institute, the patients signed an informed consent for the anonymous use of all the biochemical and anthropometric data collected during their hospitalization to be used for subsequent scientific research.

### 2.2. Measurements

#### 2.2.1. Physical Characteristics and Body Composition

The medical history and a physical examination of subjects were taken at the time of admission to the hospital. BM was measured to the nearest 0.1 kg with an electronic scale (Selus, Italy), with the subject dressed only in light underwear. Stature was measured to the nearest 0.5 cm on a standardized Harpenden stadiometer (Holtain Ltd., Crymych, Dyfed, UK). BMI was calculated as weight (kg) divided by stature (m) squared [17]. Waist circumferences (WCs) were determined in a standing position midway between the lowest rib and the top of the iliac crest after gentle expiration with a nonelastic flexible tape measure [18]. Hip circumference (HC) was measured at the greatest posterior protuberance [18]. Body composition was measured by using a multifrequency tetrapolar impedancemeter (BIA, Human-IM Scan, DS-Medigroup, Milan, Italy) with a delivered current of 800 µA at a frequency of 50 kHz. In order to reduce errors of measurement, attention was paid to the standardization of the variables that affect measurement validity, reproducibility, and precision. Measurements were performed according to the method of [19] after 20 min of resting in a supine position with arms and legs relaxed and not in contact with other body parts. FFM was calculated using the prediction equation [20], and FM was derived as the difference between BM and FFM.

#### 2.2.2. Blood Pressure Measurements

Diastolic and systolic blood pressures (BPs) were measured to the nearest 2 mmHg in the supine position after 5 min rest using a standard mercury sphygmomanometer with an appropriately sized cuff. The average of three measurements on different days was used.

### 2.3. Laboratory Analyses

Baseline blood samples were drawn by venipuncture after a 12 h overnight fast. Measurements of glycemia, high-density lipoprotein cholesterol (HDL-C), and triglycerides (TGs) were performed by standard enzymatic methods (Roche Diagnostics, Mannheim, Germany).

### 2.4. Anthropometric Indices

According to the International Diabetes Federation (IDF) criteria [3], a diagnosis of MetS is made when three or more of the following risk factors are present: a waist circumference (WC) ≥ 80 cm, fasting plasma glucose (FPG) ≥ 100 mg/dL (5.55 mmol/L) or on drug treatment for elevated glucose, systolic blood pressure (SBP) ≥ 130 mmHg or diastolic blood pressure (DBP) ≥ 85 mmHg or on antihypertensive drug treatment in a patient with a history of hypertension, fasting triglycerides (TGs) ≥ 150 mg/dL (1.7 mmol/L) or on drug treatment for elevated TG, and HDL-C < 50 mg/dL (1.3 mmol/L) or on drug treatment for reduced HDL-C.

The following indexes were calculated according to the following formulas:

WHR [21]:

WC (cm)/HC (cm)

WtHR [11]:

WC (cm)/height (cm)

BMFI [12]:

BMI (kg m^−2^) × FM (%) × WC (m)

CMI [15]:

WtHR × TG (mmol L^−1^)/HDL-C (mmol L^−1^)

VAI [14]:

(WC (cm)/36.58 + 1.89 × BMI (kg m^−2^)) × (TG (mmol L^−1^)/0.81 × 1.52/HDL (mmol L^−1^))

### 2.5. Statistical Analyses

The data (WHR, WtHR, BMFI, VAI, CMI) were first scrutinized for outliers using a cutoff of 4.5 standard deviation score. No patient was excluded on this basis. A Shapiro–Wilk test was used to assess the normality of each continuous variable. Continuous data were presented as mean and standard deviation. Anthropometric and metabolic data of women with MetS (MetS+) and without MetS (MetS−) were tested for statistical significance using two-tailed t-tests. Pearson correlation coefficients were calculated to assess the relationship between body composition indexes and SBP, DBP, TG, and HDL-C.

Receiver operating characteristic (ROC) curves were then generated to obtain the values of the area under the curve (AUC), with sensitivity, specificity, and 95% CI for each index as a predictor of MetS. In addition, for all the indexes, we calculated their sensitivity and specificity, their positive and negative predicted value (PPV and NPV, respectively), and their positive and negative likelihood ratio (PLR and NLR, respectively) to determine which index of body composition and/or adiposity was the most reliable in identifying MetS. ROC analysis was performed to identify the best predictor for each adiposity index among the metabolic risk factors. To identify the optimal cutoff, the Youden index [22] was calculated. The significance threshold was set at *p* < 0.05. The data were analyzed using STATA 17.0.

## 3. Results

### 3.1. Physical Characteristics of Subjects

A total of 876 women participated in this study. The general characteristics are shown in Table 1. According to the IDF criteria, a total of 876 women (100%) had abdominal obesity. Elevated BP was detected in 639 participants (73%). Of this total, 302 (35%) women presented an increase in TG levels, 286 (33%) showed hyperglycemia, and 498 women (57%) had reduced HDL-C levels. Overall, the presence of MetS was found in 544 patients (62%). Patients with MetS (MetS+) were significantly older than those without MetS (MetS−) (+8.20 ± 2.44 y, *p* < 0.001). Women with MetS had significantly higher values of BMI (+4%, *p* = 0.006), WC (+6%, *p* < 0.001), FFM (kg) (+2%, *p* = 0.006), SBP (+4%, *p* < 0.001), DBP (+2%, *p* = 0.006), TG (+37%, *p* < 0.001), HDL-C (−23%, *p* < 0.001), and glycemia (+22%, *p* < 0.001) than those without MetS (Table 1). MetS+ women showed higher values of WHR (+5%), WHtR (+6%), BMFI (+10%), VAI (+51%), and CMI (+53%,) than MetS− women (*p* < 0.001). No difference was found for BM, HC, FM (kg), FM (%), and FFM (%) between the two subgroups (Table 1).

### 3.2. Correlations

The correlations between the different indexes and cardiometabolic parameters are shown in Table 2. A strong correlation with MetS indexes and clinical and biochemical parameters was observed between VAI and HDL-C (R^2^ = 0.333, *p* < 0.001), VAI and TG (R^2^ = 0.827, *p* < 0.001), CMI and HDL-C (R^2^ = 0.342, *p* < 0.001), and CMI and TG (R^2^ = 0.812, *p* < 0.001).

### 3.3. ROC Curve of Anthropometric Indexes to Predict MetS

**Figure 1:**
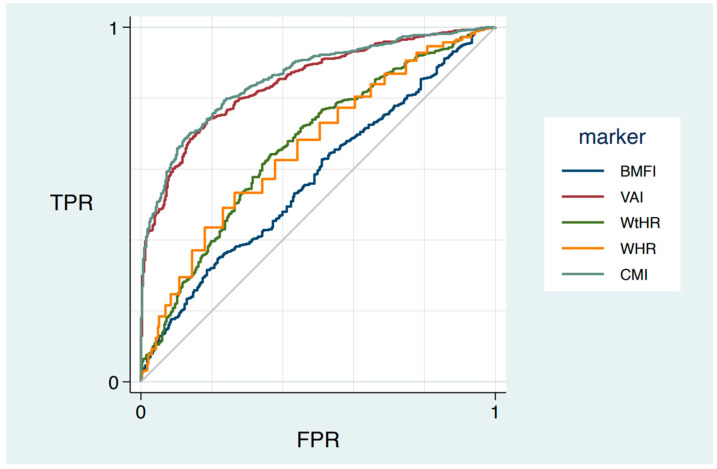
The receiver operating characteristic (ROC) curve of five anthropometric indexes in predicting metabolic syndrome among female adults. Abbreviations: WHR, waist-to-hip ratio; WtHR, waist-to-height ratio; BMFI, body mass fat index; VAI, visceral adiposity index; CMI, cardiometabolic index; TPR, true positive rate; FPR, false positive rate.

The ROC curves and the area under the curve (AUC) comparing the predictive abilities of WHR, WtHR, BMFI, VAI, and CMI for MetS in women are shown in Figure 1 and Table 3. VAI (AUC = 0.84) and CMI (AUC = 0.86) showed the best performance in predicting MetS. The optimal cutoff point for early diagnosis of MetS was >0.92 for WHR, >0.76 for WtHR, >30.1 kg m^−1^ for BMFI, >1.94 for VAI, and >0.84 for CMI. The percentages of women who would have been diagnosed with MetS using each of the indexes are the following: WHR, 35.2%; WtHR, 65.4%; BMFI, 35.2%; VAI, 66.4%; and CMI, 68.6%.

Differences were found between the ROC area of CMI and all other indexes (*p* < 0.001), VAI and all indexes (*p* < 0.002, BMFI and all indexes (*p* < 0.001), except for VAI vs. CMI (*p* = 0.002)). No difference was found between the ROC area of WHR vs. WtHR (*p* = 0.521).

Table 4 shows the sensitivity, specificity, PPV, NPV, PLR, NLR, and Youden index for identifying MetS. WtHR, VAI, and CMI were the most sensitive compared with other indexes, whereas WHR and BMFI had the lowest sensitivity. BMFI, VAI, and CMI showed the highest specificity, and WtHR was the lowest. VAI and CMI showed higher PPV and NPV compared with other indexes. PLR and the Youden index were higher for VAI and CMI compared with the other indexes, while NLR was lower for VAI and CMI than for the other indexes.

## 4. Discussion

In the present study, the accuracy of five different indexes for early identification of MetS was evaluated in a large group of women with obesity aged 18–83 years. The main findings were that VAI and CMI performed significantly better than the other indexes (i.e., WHR, WtHR, and BMFI) in detecting MetS.

First, we observed that women with MetS were older than women without MetS, and WC was higher in obese women with MetS than in women without MetS. Overall, the presence of MetS was found in 544 patients (62%). Normally, the prevalence of MetS in postmenopausal women ranges from 32 to 58% [23]. In addition, consistent with previous studies [13,16], women with MetS had a higher incidence of metabolic abnormalities, including hyperglycemia (i.e., 52%), higher TG levels (i.e., 54%), and low HDL-C (i.e., 78%). It seems plausible that increased central adiposity (i.e., WC as a marker of central adiposity) over the years, particularly after menopause [24], could contribute to increasing the likelihood of developing MetS.

Second, to our knowledge, this is the first study conducted on women with obesity that showed higher predictive accuracy in identifying MetS with two different indexes, such as VAI and CMI. These results are consistent with previous studies showing that VAI and CMI performed significantly better than the other indexes in identifying MetS in adolescents with obesity [12] and adults with obesity [16,25]. On the contrary, previous research showed that WtHR performed slightly better than the other indexes [13]. However, the study by Radetti et al. [13] did not show a direct comparison between WtHR, VAI, and CMI. Furthermore, the percentage of women who would have been diagnosed with MetS based on our cutoffs for WtHR, VAI, and CMI were 65.4%, 66.4%, and 68.6%, respectively.

Because the diagnosis of MetS involves anthropometric and biochemical parameters [3], both VAI and the CMI consider measures of body excess, fat distribution (i.e., WC and BMI), and biochemical parameters such as TG and HDL-C levels. In contrast, the WtHR only considers body composition parameters. Finally, the analysis of the correlation between the different indexes and the component of MetS confirmed that VAI and CMI were better predictors of metabolic risk factors.

Nevertheless, VAI and CMI were the most sensitive and specific indexes compared with the others. At the same time, the PPV for VAI and CMI was higher than for WHR, WtHR, and BMFI, even when NPV was considered. Overall, it was found that PPV was higher than NPV according to the prevalence of MetS in the study population. PLR and NLR were higher for the VAI and CMI indexes, suggesting that these two indexes better predict the probability of a true positive or negative outcome. In contrast to our results, Radetti et al. [13] showed that only BMI and WtHR were slightly better predictors of the probability of a positive or negative outcome, although their values were lower compared with our results. Thus, our data confirm previous observations showing that the VAI and CMI indexes have high sensitivity and specificity for detecting MetS in children and adolescents with obesity [12] and in adults with obesity [25]. Considering our results, the higher sensitivity and specificity of VAI and CMI compared with WtHR suggest that VAI and CMI may be more useful and beneficial in clinical practice to detect MetS in a cohort of women with obesity. Moreover, since people with metabolic disorders often have an accumulation of TGs and cholesterol in hepatocytes caused by an increase in visceral fat [26], MetS was also associated with an increased risk of developing nonalcoholic fatty liver disease (NAFLD) [27], as our research group recently observed in obese children and adolescents [28]. Indeed, previous studies have shown that CMI and VAI are strongly and positively associated with NAFLD risk [29]. Unfortunately, since we have not performed liver ultrasonography to diagnose NAFLD in this huge study population, this target of research will be a matter for future research.

Our study presents, however, some limitations. First, longitudinal studies are required to measure the evolution over time of the ability of the different indexes to predict MetS in women with obesity. Second, we did not perform DEXA assessment (i.e., the gold standard) of body fat in our study due to the large number of participants, the high cost, and the X-ray exposure of this measurement. The use of WC as a marker of central obesity represents a potential bias in the present study. However, the examination of the patients was performed by a well-trained and experienced team, thus reducing the risk of significant misinterpretation.

Further additional studies are needed to evaluate different indexes in the different BMI classes using DEXA in order to measure body composition in women with obesity.

Finally, we selected in the present study only Caucasian women with obesity. Therefore, it is not possible to generalize our conclusions to other ethnic groups.

On the contrary, the strength of this study lies in a large number of women with obesity who were recruited in a single third-level center for multidisciplinary obesity rehabilitation, examined by a well-trained staff of physicians, and with biochemical assessments by the same laboratory.

In summary, VAI and CMI perform better than the other indexes in detecting metabolic syndrome. The two indexes simply require the evaluation of WC, height, and BMI, and the concomitant evaluation of two simple, easy, and routinely measurable biochemical parameters. Thus, the two indexes prove to be the best predictors of MetS that can be easily used in clinical practice. However, as mentioned above, our results will require to be confirmed with the use of better methods of body composition measurement, such as DEXA. Finally, it should be emphasized that all indexes mentioned in this study should be considered a support for clinicians to identify the presence of MetS without replacing clinical assessment.

Finally, future studies are needed to determine whether changes in VAI and CMI over the years could be useful to ensure early detection of MetS and to determine the most appropriate therapeutic and rehabilitative approaches to assist clinicians in their daily clinical practice.

## Figures and Tables

**Table 1:** Descriptive statistics for all subjects, MetS−, and MetS+. Data are presented as mean ± SD. *p* is the difference between MetS− and Met+.

	All	MetS−	MetS+	*p*
Number of Subjects	876	334	542	
Age (y)	52.1 ± 13.8	47.1 ± 15.2	55.2 ± 11.8	0.001
Stature (m)	1.57 ± 0.06	1.58 ± 0.08	1.57 ± 0.07	0.108
Body mass (kg)	107.8 ± 17.1	106.4 ± 15.8	108.7 ± 17.7	0.341
BMI (kg m^−2^)	43.6 ± 6.1	42.7 ± 5.1	44.2 ± 6.6	0.006
WC (cm)	121.0 ± 12.7	116.7 ± 11.8	123.6 ± 12.5	0.001
HC (cm)	133.0 ± 13.1	132.5 ± 11.5	133.1 ± 14.0	0.982
Fat-free mass (kg)	52.2 ± 4.8	51.6 ± 4.3	52.6 ± 5.0	0.021
Fat mass (kg)	55.6 ± 14.2	54.8 ± 13.3	56.1 ± 14.7	0.761
Fat-free mass (%)	49.1 ± 5.5	49.2 ± 5.4	49.1 ± 5.5	0.967
Fat mass (%)	50.9 ± 5.5	50.9 ± 5.6	51.0 ± 5.5	0.805
SBP (mm Hg^−1^)	127.4 ± 14.3	124.0 ± 14.3	130.0 ± 13.8	0.001
DBP (mm Hg^−1^)	76.8 ± 8.0	75.8 ± 8.0	77.3 ± 7.8	0.006
TG (mg dL^−1^)	131.6 ± 63.4	96.4 ± 31.0	153.3 ± 68.4	0.001
HDL-C (mg dL^−1^)	50.1 ± 12.4	56.7 ± 12.0	46.1 ± 11.0	0.001
Glycemia (mmol L^−1^)	97.0 ± 30.5	82.2 ± 10.7	106.0 ± 35.0	0.001
WHR	0.91 ± 0.08	0.88 ± 0.07	0.93 ± 0.08	0.001
WtHR	0.77 ± 0.08	0.74 ± 0.07	0.79 ± 0.08	0.001
BMFI (kg/m)	27.6 ± 9.2	26.0 ± 7.7	28.7 ± 9.8	0.001
VAI (cm^2^)	2.44 ± 1.64	1.46 ± 0.61	3.04 ± 1.79	0.001
CMI	0.98 ± 0.65	0.58 ± 0.24	1.23 ± 0.70	0.001

All values are mean and standard deviation (SD). Abbreviations: BMI, body mass index; WC, waist circumference; HC, hip circumference; FFM, fat-free mass; FM, fat mass; SBP, systolic blood pressure; DBP, diastolic blood pressure; TG, triglyceride; HDL-C, high-density lipoprotein cholesterol; WHR, waist-to-hip ratio; WtHR, waist-to-height ratio; BMFI, body mass fat index; VAI, visceral adiposity index; CMI, cardiometabolic index.

**Table 2:** Bivariate correlation coefficients between indexes of adiposity and metabolic characteristics.

	SBP (mm Hg^−1^)	DBP (mm Hg^−1^)	HDL-C (mg dL^−1^)	Glycemia (mg dL^−1^)	TG (mg dL^−1^)
WHR	**R^2^ = 0.014**	R^2^ = 0.001	**R^2^ = 0.031**	**R^2^ = 0.064**	**R^2^ = 0.050**
	***p* = 0.001**	*p* = 0.253	***p* = 0.001**	***p* = 0.001**	***p* = 0.001**
WtHR	**R^2^ = 0.065**	R^2^ = 0.038	**R^2^ = 0.011**	**R^2^ = 0.050**	**R^2^ = 0.008**
	***p* = 0.001**	*p* = 0.253	***p* = 0.002**	***p* = 0.001**	***p* = 0.007**
BMFI (kg m^−1^)	**R^2^ = 0.040**	**R^2^ = 0.042**	**R^2^ = 0.006**	**R^2^ = 0.006**	R^2^ = 0.001
	***p* = 0.001**	***p* = 0.001**	***p* = 0.030**	***p* = 0.026**	*p* = 0.821
VAI (cm^2^)	R^2^ = 0.001	R^2^ = 0.002	**R^2^ = 0.333**	**R^2^ = 0.084**	**R^2^ = 0.827**
	*p* = 0.887	*p* = 0.631	***p* = 0.001**	***p* = 0.001**	***p* = 0.001**
CMI	R^2^ = 0.001	R^2^ = 0.001	**R^2^ = 0.342**	**R^2^ = 0.094**	**R^2^ = 0.812**
	*p* = 0.246	*p* = 0.628	***p* = 0.001**	***p* = 0.001**	***p* = 0.001**

Abbreviations: SBP, systolic blood pressure; DBP, diastolic blood pressure; TG, triglyceride; HDL-C, high-density lipoprotein cholesterol; WHR, waist-to-hip ratio; WtHR, waist-to-height ratio; BMFI, body mass fat index; VAI, visceral adiposity index; CMI, cardiometabolic index. Bold text indicates a statistically significant correlation.

**Table 3:** ROC area relating anthropometric indexes (95% CI) in all 876 women.

	ROC Area	95% Confidence Interval
WHR	0.68 °	(0.64–0.71)
WtHR	0.67 °	(0.63–0.70)
BMFI (kg m^−1^)	0.57 *	(0.54–0.61)
VAI (cm^2^)	0.84 *	(0.82–0.87)
CMI	0.86 *	(0.83–0.88)

Abbreviations: WHR, waist-to-hip ratio; WtHR, waist-to-height ratio; BMFI, body mass fat index; VAI, visceral adiposity index; CMI, cardiometabolic index. For significance: * *p* < 0.05 compared with the other indexes; ° *p* < 0.05 vs. BMFI, VAI, and CMI.

**Table 4:** Sensitivity, specificity, positive predictive value, negative predictive value, positive likelihood ratio, negative likelihood ratio, and Youden index for each anthropometric index in identifying metabolic syndrome (MetS).

	Sensitivity	Specificity	PPV	NPV	PLR	NLR	Youden Index
WHR	57.2%	65.8%	73.1%	48.6%	1.67	0.62	0.27
WtHR	64.2%	63.1%	74.0%	52%	1.74	0.57	0.28
BMFI (kg m^−1^)	34.7%	78.1%	72%	42.3%	1.58	0.84	0.13
VAI (cm^2^)	73.8%	81.4%	86.6%	65.6%	3.96	0.32	0.55
CMI	68.6%	87.7%	90.1%	63.2%	5.57	0.36	0.56

Abbreviations: WHR, waist-to-hip ratio; WtHR, waist-to-height ratio; BMFI, body mass fat index; VAI, visceral adiposity index; CMI, cardiometabolic index; PPV, positive predictive value; NPV, negative predictive value; PLR, positive likelihood ratio; NLR, negative likelihood ratio.

## Data Availability

Raw data will be available on 24 April 2023 upon a reasonable request to the corresponding author, and they will be uploaded on www.zenodo.org immediately after the acceptance of the manuscript.
